# Warming the Intrinsic Foot Muscles: A Specific Warm-Up for Dynamic Stability in a Pilot Study

**DOI:** 10.7759/cureus.91939

**Published:** 2025-09-09

**Authors:** Yutaro Hyodo, Takumi Jiroumaru, Hikaru Kitagawa, Takamitsu Fujikawa

**Affiliations:** 1 Department of Physical Therapy, Bukkyo University, Kyoto, JPN; 2 Rehabilitation, Kanazawa Orthopaedic &amp; Sports Medicine Clinic, Shiga, JPN

**Keywords:** dynamic balance, intrinsic foot muscles, neuromuscular control, sports injuries, star excursion balance test, thermal intervention

## Abstract

Background

Optimizing the intrinsic foot muscles (IFMs) is a key aspect of athletic conditioning for enhancing dynamic balance and preventing injury. However, conventional warm-up strategies often neglect the IFMs in favor of proximal muscle groups, leaving their specific contribution under-investigated. This randomized crossover pilot study primarily tested whether localized warming of the IFMs using capacitive and resistive electric transfer (CRET) improves dynamic balance on the modified Star Excursion Balance Test (mSEBT) compared with warming the posterior calf. Secondary objectives were to assess changes in deep tissue temperature and weight-bearing ankle dorsiflexion.

Methods

Nine healthy adult males participated in this randomized crossover trial. Participants received a two-minute CRET intervention applied to the IFMs, and in a separate session, after a one-week washout period, to the posterior calf muscles. The primary outcome was the normalized reach distance in the three directions (anterior (ANT), posteromedial (PM), and posterolateral (PL)) of the mSEBT. Secondary outcomes included deep tissue temperature and ankle dorsiflexion range of motion, measured with the weight-bearing lunge test (WBLT). Data were analyzed using nonparametric tests.

Results

Deep tissue temperature and ankle dorsiflexion (WBLT) significantly increased after both the IFM and posterior calf interventions. However, a significant and selective improvement in mSEBT performance was observed only in the PL reach distance following the IFM intervention (p=0.029). The posterior calf intervention did not yield a significant improvement in the PL direction. No significant changes were found in the ANT or PM directions under either condition.

Conclusions

IFMs are a viable target for pre-activity thermal interventions. The selective improvement in the PL direction suggests that heating the plantar foot enhances the neuromuscular control required for challenging, multiplanar balance tasks, an effect that extends beyond simple increases in flexibility. This finding implies that our intervention specifically benefited the foot and ankle complex, which is heavily taxed in the PL direction. This approach offers a focused warm-up strategy to optimize performance and potentially mitigate injury risks.

## Introduction

Intrinsic foot muscles (IFMs) play a crucial role in actively supporting the longitudinal and transverse arches of the foot and controlling body balance [1,2]. To control dynamic balance, mechanoreceptors in the plantar sole and muscle spindles within the IFMs serve as the primary sources of sensory input for detecting postural changes [3,4]. This sensory feedback enables IFMs to contract rapidly, enhancing ankle stability and thereby maintaining body balance. Evidence shows that toe flexor strength is negatively correlated with fall risk in older adults [5] and that IFM thickness is positively correlated with single-leg stance postural control [6]. These findings suggest that the IFMs are essential for balance control.

Dynamic balance is defined as the ability to maintain postural stability during movement. In sports such as soccer and basketball, which demand sudden changes in direction and jump landing, significant stress is placed on the knee and ankle joints. Poor dynamic balance increases the risk of sports injuries, including anterior cruciate ligament rupture and ankle sprains [7,8]. Pre-activity warm-ups are an effective strategy for reducing injury risk and enhancing performance [9]. Warm-ups increase muscle temperature, which optimizes neuromuscular function by improving nerve conduction velocity and accelerating metabolic reactions within the muscle [10]. This leads to greater efficiency and speed of force production. While this effect has traditionally been achieved through systemic exercises like jogging, approaches using physical modalities have recently gained attention. For instance, one study reported that Capacitive and Resistive Electric Transfer (CRET) therapy increased local muscle temperature and improved jump performance [11]. These findings suggest that local muscle warming may be a viable strategy for enhancing dynamic balance.

The Star Excursion Balance Test (SEBT) is widely used for assessing dynamic balance. The original eight-direction test has been shown to be simplified to three directions (anterior (ANT), posteromedial (PM), and posterolateral (PL)) without compromising its reliability [12]. This modified SEBT (mSEBT) reduces testing time and is therefore widely used in both clinical and research settings for functional assessment after injuries such as ACL ruptures and ankle sprains, as well as for injury risk prediction [7,12,13].

Recent biomechanical studies have revealed that each reach direction of the mSEBT reflects a different neuromuscular control strategy [14]. The ANT and PM directions are primarily influenced by the force-producing capacity of the proximal muscles around the hip and knee. By contrast, the PL direction places greater demands on the coordinated control of the entire lower extremity, including the foot and ankle complex. However, previous research has primarily focused on the function of these proximal segments. Consequently, the effect of interventions targeting the foot and ankle, the foundation of dynamic balance, and specifically the IFMs, on mSEBT performance has not been adequately investigated.

Therefore, the primary purpose of this pilot study was to investigate whether a targeted CRET thermal intervention on the IFMs would improve dynamic balance performance (mSEBT) compared to an intervention on the posterior calf muscles. The secondary objectives were to assess changes in deep tissue temperature and ankle dorsiflexion. We hypothesized that the CRET therapy applied to the IFMs would improve mSEBT reach distances more significantly than the intervention applied to the posterior calf. The findings of this study could provide new evidence for the importance of foot-focused approaches in warm-up strategies aimed at preventing sports injuries.

## Materials and methods

Participants

Nine healthy adult males (age: 31.2±6.9 years, height: 172.0±10.6 cm, body mass: 67.9±9.5 kg) participated in this study. The exclusion criteria were as follows: (1) history of orthopedic injury to the lower extremities within the past six months, (2) any dermatological conditions, or (3) any metallic implants.

This study was approved by the Kanazawa Orthopedic Surgery and Medical Clinic Ethics Committee (Approval No: Kanazawa-OSMC-2024-001). The study protocol was registered with the University Hospital Medical Information Network (UMIN) Clinical Trials Registry (Registration No: UMIN000058301). The purpose and procedures of the study were explained to all participants, both orally and in writing, and written informed consent was obtained from each participant. Data collection for this study was conducted from July 1, 2025, to August 1, 2025.

Experimental procedure

This study employed a randomized crossover design. All participants first underwent baseline measurements. Subsequently, they were randomly assigned to an intervention sequence (posterior calf first or plantar foot first). The randomization sequence was generated by a researcher not involved in participant recruitment, using a computer-based random number generator. Allocations were concealed in sequentially numbered, opaque, sealed envelopes. As each participant was enrolled, the next available envelope was opened to reveal their group assignment. A counterbalanced approach was thus applied to offset any order-related effects from the interventions. A one-week washout period was scheduled between each intervention session to eliminate any carryover effects. While the participants and the clinician administering the CRET could not be blinded, the investigator who performed all outcome measurements was blinded to the intervention condition for each session.

Intervention

A CRET device (Physio Radio-Stim Pro; Sakai Medical Co., Ltd., Tokyo, Japan) was used for the intervention. The capacitive (CET) mode of the CRET was used with a circular probe with a diameter of 60 mm at a frequency of 500 kHz. A large rectangular return plate (200 x 210 mm) was secured over the belly of the tibialis anterior muscle, serving as the inactive electrode. Participants were placed in the prone position. After applying a specialized cream to the electrode and skin surface, a two-minute thermal intervention was administered at one of the two sites.

For the posterior calf condition, the intervention targeted the gastrocnemius and soleus muscles, applied over an area from the popliteal fossa to the Achilles tendon insertion. For the plantar foot condition, the intervention targeted the IFMs applied over an area from the calcaneus to the metatarsal heads.

During the intervention, the clinician maintained the probe in constant motion. The intensity was individually adjusted to the level at which the participant felt comfortable warmth (approximately 7/10 on the Visual Analog Scale) to normalize the perceptual thermal stimulus across participants.

Measurements

All outcome measures were recorded immediately after each intervention.

Deep tissue temperature

Immediately before and after the intervention, the deep tissue temperature was measured using a zero-heat-flux deep thermometer (Coretemp CTM-205, Terumo Corporation, Tokyo, Japan). The probe was secured with tape over the center of the medial gastrocnemius muscle belly in the posterior calf condition and at the midpoint of a line connecting the center of the calcaneal tuberosity and the third metatarsal head in the plantar foot condition.

Weight-bearing dorsiflexion range of motion

The weight-bearing lunge test (WBLT) was used to measure ankle dorsiflexion and its range of motion. The participants stood facing a wall with the center of their heel and first toe aligned with a tape measure on the floor. From this position, they were instructed to move their knee forward toward the wall while keeping their heel on the floor. The maximum distance (cm) from the tip of the first toe to the wall, where the knee touched the wall, was recorded.

Modified Star Excursion Balance Test (mSEBT)

The participants stood on their dominant leg to measure their reach distance in three directions: ANT, PM, and PL. The PM and PL lines were marked on the floor at an 135° angle relative to the ANT line. Participants were instructed to lightly touch the floor with the tip of their reaching foot and were informed that a trial would be considered invalid if they lost their balance. The average of the three valid trials was calculated after several practice trials in each direction. The order of the directions was randomized. The reach distances were normalized to leg length (%). The leg length was measured as the distance from the anterior superior iliac spine to the inferior aspect of the medial malleolus.

Statistical analysis

All statistical analyses were performed using IBM SPSS Statistics for Windows, Version 28 (Released 2021; IBM Corp., Armonk, New York, United States). The level of statistical significance was set at p<0.05. First, the Shapiro-Wilk test was used to assess all data for normality. Because some data were not normally distributed, nonparametric tests were performed. The Friedman test was used to compare the three conditions (baseline, posterior calf intervention, and plantar foot intervention). If a significant difference was found, the Wilcoxon signed-rank test with Bonferroni correction was performed as a post-hoc test. The Wilcoxon signed-rank test was used to compare the pre- and post-intervention temperature changes. The relationship between the variables was evaluated using Spearman's rank correlation coefficient (ρ). Effect sizes were calculated using Kendall's W for the Friedman test and the r-value for the Wilcoxon signed-rank test. Demographic data are presented as mean ± standard deviation, while all other descriptive statistics are presented as median and interquartile range (IQR).

## Results

Data from all nine participants were analyzed. The demographic and physical characteristics of the participants are presented in Table 1.

**Table 1:** Baseline characteristics of the participants Values are presented as mean ± standard deviation.

Variable	Total (n=9)
Age (years)	31.2±6.9
Height (cm)	172.0±10.6
Weight (kg)	67.9±9.5

Descriptive statistics for all outcome measures for each condition are presented in Table 2.

**Table 2:** Median (IQR) values for all outcome measures at baseline and after each intervention (n=9) Values are presented as median (Interquartile range or IQR); *p<0.05 vs. baseline (result of post-hoc test with Bonferroni correction); ^‡^p<0.01 vs. pre-intervention for each respective site (result of Wilcoxon signed-rank test); CRET, Capacitive and Resistive Electric Transfer; WBLT, Weight-Bearing Lunge Test; Temp, Temperature; mSEBT, Modified Star Excursion Balance Test; ANT, anterior; PM, posteromedial; PL, posterolateral.

Variable	Baseline	Post-posterior calf CRET	Post-plantar foot CRET	p-value (Friedman)
WBLT (cm)	13.1 (4.2)	13.5 (4.1)^*^	14.3 (3.8)^*^	<0.001
Posterior posterior calf temp (°C)	32.6 (1.7)	35.9 (1.8)^‡^	-	-
Plantar foot temp (°C)	31.3 (1.5)	-	31.8 (1.5)^‡^	-
mSEBT ANT (%)	107.1 (16.2)	105.0 (15.3)	107.2 (12.5)	0.097
mSEBT PM (%)	112.6 (9.5)	115.5 (14.9)	115.8 (11.7)	0.549
mSEBT PL (%)	113.4 (11.1)	113.3 (10.8)	114.6 (12.1)^*^	0.032

To confirm the effect of the thermal intervention, deep tissue temperatures were compared before and after the intervention. The Wilcoxon signed-rank test showed that the deep tissue temperature significantly increased post-intervention in both the posterior calf (Z=2.666, p=0.008, r=0.63) and plantar foot conditions (Z=2.675, p=0.007, r=0.63), with large effect sizes for both.

For the WBLT, the Friedman test revealed a significant difference in dorsiflexion reach distance among the three conditions (χ²(2) =13.941, p<0.001), with a large effect size (W=0.775). Post-hoc analysis showed that, compared to baseline, there was a significant increase in reach distance following both the posterior calf condition (adjusted p=0.040) and the plantar foot condition (adjusted p=0.001).

For the mSEBT, the results for the reach distance varied with direction. In the PL direction, a significant difference was found among the three conditions (χ²(2) =6.889, p=0.032, W=0.383). Post-hoc testing revealed that only the plantar foot condition showed a significant increase in reach distance compared with baseline (Z=−2.593, adjusted p=0.029, r=0.86). There was no significant difference between the posterior calf condition and the baseline (adjusted p=1.000). In contrast, no significant differences among the conditions were found for the ANT direction (χ²(2) =4.667, p=0.097, W=0.259) or the PM direction (χ²(2) =1.200, p=0.549, W=0.067).

The results of the correlation analysis between the change in the mSEBT PL reach distance and changes in other metrics are shown in Table 3.

**Table 3:** Spearman's correlation coefficients (ρ) between the change in mSEBT PL and other variables Values represent Spearman's rank correlation coefficient (ρ) and the p-value; Δ (delta), change score (post-intervention value minus baseline value); mSEBT, Modified Star Excursion Balance Test; PL, posterolateral; Temp, Temperature; WBLT, Weight-Bearing Lunge Test.

	Δ mSEBT PL
Δ Plantar foot temp	ρ=0.561, p=0.116
Δ WBLT	ρ=0.400, p=0.286

A moderate, though not statistically significant, positive correlation was observed between the change in PL reach distance and the change in plantar foot deep tissue temperature (ρ=0.561, p=0.116). Furthermore, no significant correlation was found between the change in PL reach distance and the change in WBLT reach distance (ρ=0.400, p=0.286).

## Discussion

The main finding of this pilot study was that a targeted thermal intervention on the IFMs selectively improved performance in the posterolateral direction of the mSEBT, which partially supported our initial hypothesis. Specifically, the reach distance in the PL direction of the mSEBT significantly and selectively increased after the IFM intervention. However, the reach distances in the ANT and PM directions did not change despite an increase in the ankle dorsiflexion range of motion. This finding does not align with our hypothesis regarding the ANT and PM directions. This discrepancy suggests that the relationship among IFM function, ankle mobility, and dynamic balance is more complex and direction-dependent than previously assumed.

The selective improvement in the PL reach distance suggests that the intervention influenced sensorimotor mechanisms, specifically the neuromuscular function of the IFMs, beyond a purely mechanical effect (i.e., increased flexibility). Increased muscle temperature is known to accelerate nerve conduction velocity, which can lead to faster muscle reaction times and heightened sensitivity to sensory receptors such as muscle spindles. This aligns with the findings of Taş et al. (2020), who suggested that superior balance is associated with enhanced neuromuscular function, such as improved muscle spindle sensitivity and faster muscle reaction times [15]. Therefore, we propose that enhanced proprioceptive feedback and a more rapid motor response induced by warming are the primary factors that improve control during multiplanar PL reach. This interpretation is supported by previous research showing that IFM training improves dynamic balance through enhanced neuromuscular control [16]. The thermal intervention used in this study may have enhanced the ability to make rapid and precise adjustments to maintain foot stability during the execution of a hip-driven dynamic task.

The lack of change in the ANT and PM reach distances can be attributed to the different factors that determine the performance in each direction. According to biomechanical data from Nelson et al. (2021), performance in the ANT and PM directions is limited primarily by the force-producing capacity of the proximal muscle groups around the hip and knee, which requires large knee extension, hip extension, and hip abduction moments for success [14]. In contrast, the PL direction places the greatest demand on the foot and ankle complex for motor control in the frontal and transverse planes. In fact, the PL direction has been reported to have a higher rate of failed attempts in athletes than other directions, suggesting that it is the most challenging for balance control [17]. Therefore, it is likely that our intervention not only increased passive ankle flexibility but also enhanced neuromuscular control of the foot, enabling participants to meet the heightened stability demands of the PL direction.

The findings of this study have important practical implications for athletic conditioning, rehabilitation, and pre-activity. While comprehensive injury prevention programs are effective in improving general performance factors such as balance and strength, targeted strategies to address specific functional deficits are equally important [18,19]. Our results may offer a new approach for addressing dynamic balance asymmetry, which is a common clinical issue. For example, incorporating local thermal intervention targeting the IFMs into a warm-up routine could be a specific strategy for correcting such imbalances. Interventions in the plantar foot, an area rich in muscle spindles and sensory receptors, may enhance body awareness. This can help athletes refine their movement patterns and achieve improved neuromuscular control.

The results of this study should be interpreted with consideration of several limitations. First, as a pilot study, the small sample size (n=9) limits the statistical power and the generalizability of our findings. While our results provide a promising signal, these findings should be considered preliminary and hypothesis-generating rather than definitive grounds for immediate changes in clinical practice. Second, our sample consisted exclusively of healthy young males, and the results may not be generalizable to female participants or other age groups. Third, while our intervention improved performance in the PL direction, it did not affect the ANT or PM directions. Therefore, our conclusions are specific to this challenging component of dynamic balance and should not be interpreted as a general improvement in all aspects of dynamic balance. Fourth, this study only investigated acute effects; it remains unknown whether these performance improvements translate to a reduction in actual injury rates over a competitive season. Finally, the proposed neuromuscular mechanism is inferential, as we did not directly measure post-intervention toe flexor strength, the strength of key proximal muscles such as the gluteus maximus or quadriceps, or changes in neuromuscular control via electromyography (EMG) during weight-bearing tasks. Future large-scale studies are recommended to validate the findings of this study and incorporate muscle strength assessments and electromyographic measurements. This would help clarify the specific effects of a foot-focused intervention on foot muscle strength and neural control, in contrast to the contributions of proximal muscles.

## Conclusions

This pilot study demonstrated that a CRET thermal intervention on the IFMs selectively improved performance in the PL direction of the mSEBT. In contrast, performance did not improve in the ANT or PM directions, which rely more on proximal muscle strength, despite a general increase in ankle flexibility. These findings suggest that targeted thermal intervention of the foot enhances the specific neuromuscular control of the foot-ankle complex required for challenging dynamic balance tasks.

This approach may serve as a novel and focused warm-up strategy. By specifically targeting the IFMs, clinicians and trainers can potentially address deficits in the posterolateral component of dynamic balance, which is crucial for multiplanar sports. Future large-scale studies are warranted to validate these findings in diverse populations and to determine if this acute performance enhancement translates to a long-term reduction in injury risk.
